# Clinical Features Observed in General Practice Associated With the Subsequent Diagnosis of Progressive Supranuclear Palsy

**DOI:** 10.3389/fneur.2021.637176

**Published:** 2021-04-22

**Authors:** Mary J. Kwasny, Denise M. Oleske, Jorge Zamudio, Robert Diegidio, Günter U. Höglinger

**Affiliations:** ^1^Department of Preventive Medicine, Feinberg School of Medicine, Northwestern University, Chicago, IL, United States; ^2^Global Epidemiology, AbbVie Inc., North Chicago, IL, United States; ^3^Global Medical Affairs, AbbVie Inc., North Chicago, IL, United States; ^4^Department of Neurology, Hannover Medical School, Hannover, Germany; ^5^German Center for Neurodegenerative Diseases (DZNE), Munich, Germany

**Keywords:** progressive supranuclear palsy, machine learning, case-control study, epidemiology, electronic medical records, general practice

## Abstract

**Background:** Progressive supranuclear palsy (PSP) is a rare neurodegenerative disorder that is difficult for primary care physicians to recognize due to its progressive nature and similarities to other neurologic disorders. This case-control study aimed to identify clinical features observed in general practice associated with a subsequent diagnosis of PSP.

**Methods:** We analyzed a de-identified dataset of 152 PSP cases and 3,122 matched controls from electronic medical records of general practices in Germany. We used a random forests algorithm based on machine learning techniques to identify clinical features (medical conditions and treatments received) associated with pre-diagnostic PSP without using an *a priori* hypothesis. We then assessed the relative effects of the features with the highest importance scores and generated multivariate models using clustered logistic regression analyses to identify a subset of clinical features associated with subsequent PSP diagnosis.

**Results:** Using the random forests approach, we identified 21 clinical features associated with pre-diagnostic PSP (odds ratio ≥2.0 in univariate analyses). From these, we constructed a multivariate model comprising 9 clinical features with ~90% likelihood of identifying a subsequent PSP diagnosis. These features included known PSP symptoms, common misdiagnoses, and 2 novel associations, diabetes mellitus and cerebrovascular disease, which are possible modifiable risk factors for PSP.

**Conclusion:** In this case-control study using data from electronic medical records, we identified 9 clinical features, including 2 previously unknown factors, associated with the pre-diagnostic stage of PSP. These may be used to facilitate recognition of PSP and reduce time to referral by primary care physicians.

## Introduction

Progressive supranuclear palsy (PSP) is a rare neurodegenerative movement disorder, with an age-adjusted prevalence rate of 5–7 per 100,000 (1). Twelve diagnostic clinical features associated with PSP have been identified in the 2017 Movement Disorder Society criteria and can be grouped under 4 functional domains: ocular motor dysfunction, postural instability, akinesia, and cognitive dysfunction (2). However, due to its progressive nature, various clinical phenotypes, and similarities to other neurologic disorders, the clinical diagnosis of PSP is complex (1). Currently, an autopsy to identify the characteristic aggregation of 4-repeat tau proteins in the basal ganglia and brainstem remains the gold standard for a definite PSP diagnosis (2, 3).

Progressive supranuclear palsy shares many overlapping clinical symptoms and features with Parkinson's disease, especially at the early symptomatic stage of PSP, and it may be difficult to differentiate between the 2 even with the use of neuroimaging techniques (4–7). This may lead to delayed diagnosis or misdiagnosis of PSP and hinder early intervention to slow disease progression. Therefore, there is a need to identify a set of clinical features associated with early PSP that can be easily recognized in general practice and lend practical insights to general practitioners, internists, and neurologists.

While previous studies have examined clinical features associated with the pre-diagnostic stage of PSP, they have been limited by small sample sizes and lack of comparison populations (3, 8, 9). Electronic medical records (EMR) are large patient databases that represent real-world practice patterns and include a myriad of clinical factors, thus they are a fertile resource for identifying potential risk factors associated with rare diseases. By applying machine learning techniques, it is possible to compare large numbers of characteristics between cases and matched controls identified from EMRs without using *a priori* hypotheses, allowing for an unbiased investigation.

In this study, our primary objective was to identify clinical features and treatments administered in the pre-diagnostic phase, as reported by general practitioners and internists in EMRs, associated with a subsequent diagnosis of PSP. Our secondary objectives were to identify new diagnostic markers, potential etiologic factors, and possible modifiable risk factors for PSP among these clinical features.

## Methods

### Study Design and Inclusion Criteria

**Figure 1:**
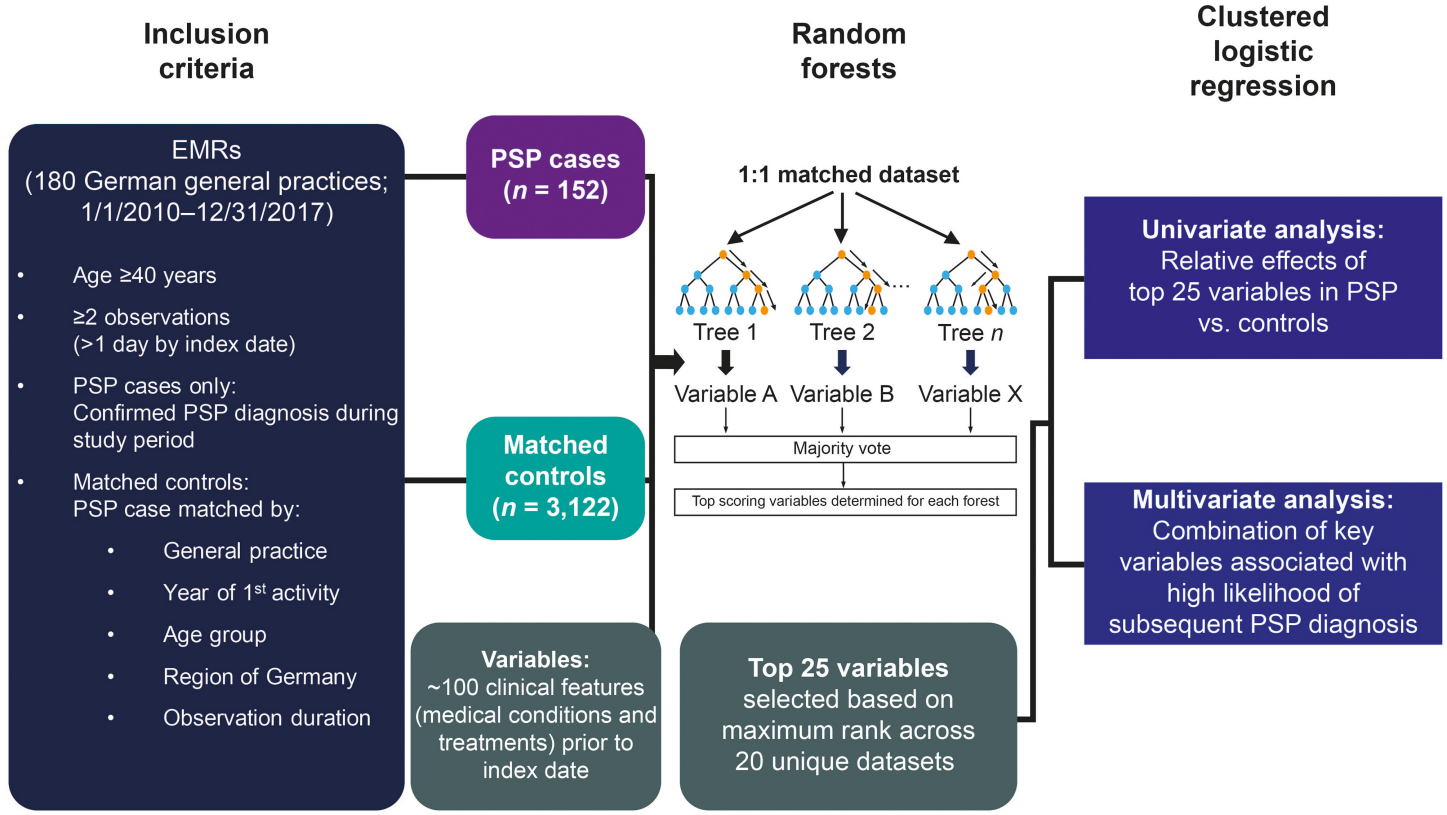
Study design using a machine learning approach. EMR, electronic medical record; PSP, progressive supranuclear palsy.

De-identified patient data were obtained from the EMRs (Disease Analyzer, IQVIA) of general practitioners and internists from 180 practices across Germany over the period from January 1, 2010 to December 31, 2017 (Figure 1). Progressive supranuclear palsy cases included patients with a first diagnosis of PSP (as defined by ICD-10 G23.1) and then with a status “confirmed” PSP. That is, the PSP diagnosis was recorded at least twice; first by the internist or general practitioner (GP), and then recorded from the diagnosis made by a consulting neurologists. The index date is defined as the date on which the PSP status was confirmed. To assign matched controls, each PSP case was matched with a random sample of 100 non-PSP patient records from the same practice. All controls had at least 1 visit within the study period and were further matched to cases based on year of first database activity, age group, region of residence (Eastern or Western Germany), and observation length. Individuals aged ≥40 years and with an observation period of >1 day by the index date of the matched case were included. Medical conditions and prescriptions recorded in the EMR prior to the index date were compared between cases and matched controls using the random forests machine learning technique to identify any clinical features associated with subsequent PSP diagnosis.

### Variables

Clinical features assessed included all medical conditions and prescriptions recorded in the eligible patient's EMR prior to the index date. For medical conditions, individual diagnosis codes were reviewed by 3 physicians [a neurologist (GUH), an internal medicine specialist (JH), and a general medical physician (JZ)] and assigned to a grouping based on an ICD-10 code (DMO). Prescriptions for drugs and medical supplies in the database were coded according to the World Health Organization Anatomical Therapeutic Chemical Classification system to the 5th digit level.

### Random Forests Analyses

We used a random-penalized conditional logistic regression algorithm package for R to evaluate the relative importance of ~100 variables included from the EMRs (10). This algorithm is applicable for the analysis of high dimensional data from 1:1 matched case-control studies. To minimize potential biases from class imbalance in the sample sizes, 10 unique 1:1 matched datasets were randomly created from the full dataset. For each dataset, 15, 20, and 25 bootstrapped trees were drawn, which considered 6, 8, or 10 randomly selected factors, respectively. These various scenarios were considered to maximize the likelihood that each variable would be assessed multiple times. Variables were scored based on relative importance, the top 5 scoring variables were determined for each forest, and the maximum rank for each variable across the 10 datasets was calculated.

### Logistic Regression Analyses

The 25 features with the highest importance scores were selected as potential clinical features associated with PSP. We compared the prevalence of these clinical features in PSP cases and controls and conducted univariate analyses to assess the relative effects of these features. Using this subset of clinical features, we randomly divided the complete dataset into training (80%) and validation (20%) datasets, stratified by matched case sets. Clustered logistic regression models with backward elimination were fit to the training dataset to determine the best-fitting multivariate model. We developed 2 multivariate models, based on the exclusion or inclusion of prior diagnosis of Parkinson's disease as a variable, respectively. Sex (male/female) was added as a variable in the model for conceptual epidemiologic reasons, although it was not significant in the univariate analysis and there is no evidence that PSP is influenced by sex. Odds ratios (ORs) and 95% confidence intervals (CIs) are shown for the selected model variables. The models determined by the training dataset were then fit with the validation dataset, and the area under the curve (AUC) was calculated from the receiver operating characteristic curve. All matching, descriptive analyses, graphic construction, and conditional logistic regression analyses were performed using SAS v9.4 (Cary, NC).

## Results

### Patients

A total of 226 PSP cases and 18,000 controls were identified in the database. After selecting for age (≥40 years), confirmed PSP diagnosis, index date (after 2010), and number of visits (≥2 visits >1 day prior to PSP diagnosis), 152 PSP cases were included in the final sample population (Figure 1 and Supplementary Table 1). Each PSP case was matched to an average of 20.5 controls (range 1–92) from the same clinic and adjusted for index date, age, region, and duration of observation in the database, resulting in a total of 3,122 matched controls in the final sample.

Overall, the matching procedure yielded controls that had similar demographics (age, sex, and region) to the cases (Supplementary Table 2). Most patients were from the western region of Germany and the mean age was ~73. The average time in the database was 5.9 years for PSP cases prior to diagnosis and 7.5 years for the controls prior to their respective matched case. Patients with PSP had more frequent hospitalizations, which is often considered to be a proxy measure of “illness” level.

### Clinical Features Identified With the Random Forests Approach Associated With Subsequent PSP Diagnosis

**Figure 2:**
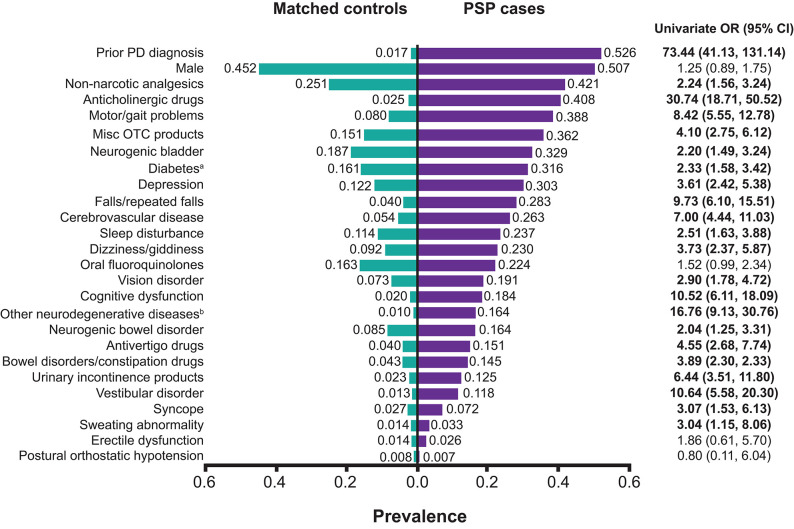
Prevalence of clinical factors during the pre-diagnostic phase by PSP case and control status. ^a^Including both type 1 and type 2 diabetes. Among PSP cases with diabetes, 8.3% had type 1 diabetes only, 87.5% had type 2 diabetes only, and 4.2% had both type 1 and type 2 diabetes. All of the matched controls had type 2 diabetes, including 0.6% who had both type 1 and type 2 diabetes. ^b^Neurodegenerative diseases other than PD or PSP. CI, confidence interval; misc, miscellaneous; OR, odds ratio (values in bold letters indicate statistically significant associations with PSP status); OTC, over-the-counter; PD, Parkinson's disease; PSP, progressive supranuclear palsy.

Using the random forests algorithm, we identified 25 clinical features with the highest importance scores including observed symptoms, clinical conditions, and treatments received during the pre-diagnostic phase as reported by general practitioners or internists associated with PSP cases. The prevalence of each clinical feature in PSP cases vs. controls prior to the index date is shown in Figure 2.

Symptoms reported in the EMRs associated with pre-diagnostic PSP included falls/repeated falls, sleep disturbance, dizziness/giddiness, and cognitive dysfunction. The most prevalent clinical conditions among patients with PSP prior to their diagnosis compared with controls were prior diagnosis of Parkinson's disease (52.6 vs. 1.7%), motor problems (38.8 vs. 8.0%), neurogenic bladder (32.9 vs. 18.7%), diabetes (31.6 vs. 16.1%), and depression (30.3 vs. 12.2%) (Figure 2). Both type 1 and type 2 diabetes (diabetes mellitus) were included in our analysis, although >90% of those with diabetes were type 2 diabetes across both PSP cases and controls.

In terms of treatments prescribed prior to their PSP diagnosis, more patients with subsequent PSP diagnosis were treated with prescription analgesics, followed by anticholinergic drugs, oral fluoroquinolones, antivertigo drugs, and drugs for bowel disorders/constipation, compared with matched controls (Figure 2). Although more patients with PSP used “miscellaneous over-the-counter products” compared with controls, we did not consider this variable to be associated with a subsequent PSP diagnosis because of the wide variety of different products in this group. However, we noted that the prevalence of outpatient resource utilization in this category among patients with PSP was more than 2-fold higher than controls.

The majority of these clinical features (21 out of 25) identified using the random forests approach, except erectile dysfunction, postural orthostatic hypotension, and the use of oral fluoroquinolones, had odds ratios of ≥2 and were associated with PSP cases according to univariate analyses. However, the variables male gender, oral fluoroquinolones, and erectile dysfunction all had odds ratios suggestive of a positive association with PSP but were not statistically significant. The variable miscellaneous over the counter products comprised a wide variety of items such as wound dressings, urinary catheters, etc. Although identified from machine learning as important, it was not included in the modeling due to its lack of specificity (Figure 2).

### Clinical Features Associated With Subsequent PSP Diagnosis, Verified Through Multivariate Models

**Figure 3:**
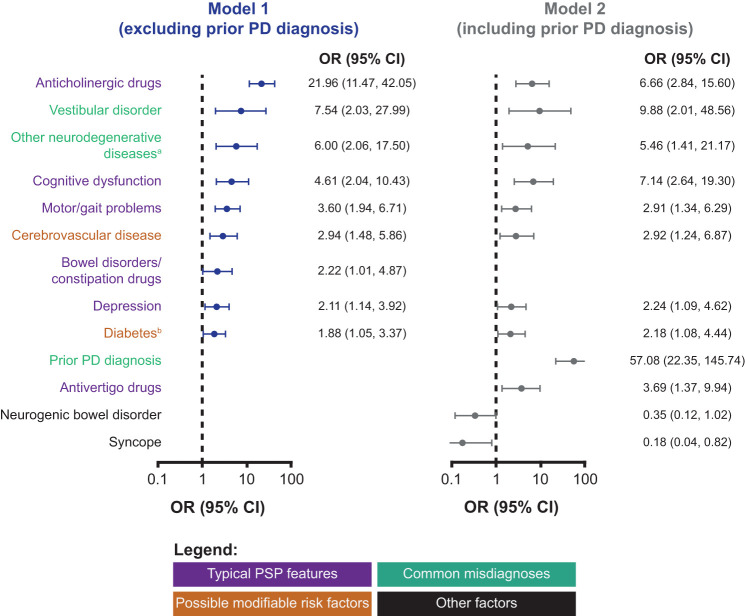
Relative effects of clinical features associated with subsequent PSP diagnosis according to multivariate models. ^a^Neurodegenerative diseases other than PD or PSP. ^b^Including both type 1 and type 2 diabetes, although the majority of patients with PSP had diabetes mellitus (see Figure 2). CI, confidence interval; OR, odds ratio; PD, Parkinson's disease; PSP, progressive supranuclear palsy.

From the clinical features identified using the random forests approach, we further found that 9 of the clinical features reported by general practitioners and internists were associated with a subsequent PSP diagnosis in a multivariate model when prior diagnosis of Parkinson's disease was excluded as a variable. These were, in order of decreasing relative effect, treatment with anticholinergic drugs, vestibular disorders, neurodegenerative disease other than Parkinson's disease or PSP, cognition problems, motor/gait problems, cerebrovascular disease, drugs for bowel disorders/constipation, depression, and diabetes (Figure 3).

When this combination of clinical features is observed in a patient in general practice, this may indicate a high likelihood of identifying a PSP case (AUC: 88%; 95% CI: 85–91%), which has also been confirmed by the validation dataset (AUC: 90%; 95% CI: 85–95%; Supplementary Figure 1A).

Most of the clinical features observed in patients with pre-diagnostic PSP in general practice were consistent regardless of whether prior diagnosis of Parkinson's disease was included or excluded as a variable (Figure 3). A total of 12 clinical features were included in the model with prior diagnosis of Parkinson's disease (PD) as a variable, and this combination of features is also associated with a high likelihood of identifying a PSP case using the training (AUC: 91%, 95% CI: 88–94%) and validation (AUC: 90%, 95% CI: 83–97%) datasets. In this model, prior diagnosis of Parkinson's disease was highly associated with subsequent PSP diagnosis (OR: 57.08; 95% CI: 22.35–145.74), while the use of antivertigo drugs but not drugs for bowel disorders/constipation was associated with PSP. In addition, PSP cases with prior PD diagnosis were associated with a lower likelihood of syncope and neurogenic bowel disorder.

## Discussion

In this report, we have presented the results of the first matched case-control study to evaluate clinical features reported in general practice EMRs associated with a subsequent diagnosis of PSP. Using the random forests machine learning approach, we identified 21 clinical features associated with pre-diagnostic PSP. We subsequently refined this subset of features to 9 clinical features using multivariate modeling, which when observed together in a patient indicated a high probability of a subsequent diagnosis of PSP. These included known clinical features associated with PSP (motor/gait problems, cognitive dysfunction, and depression); treatments related to common symptoms associated with PSP (anticholinergic drugs and drugs for bowel disorders/constipation); possible misdiagnoses (neurodegenerative disease other than Parkinson's disease or PSP and vestibular disorders); and 2 possible modifiable risk factors for PSP, cerebrovascular disease and diabetes mellitus.

Recognizing this pattern of clinical features in a patient may help primary care physicians identify possible PSP cases and refer these patients to specialists. This will become more important with the availability of disease-modifying therapies that will slow disease progression in early PSP (11).

### Typical Clinical Features of PSP

Gait/motor problems (specifically akinesia) and cognitive dysfunction are both core clinical domains of PSP diagnosis (2). Depression is also frequently observed in patients with PSP, even at the early stages (9, 12). Consistent with our findings, a recent case-control study based on patient and caregiver interviews reported that gait problems and depression were commonly detected >3 years prior to PSP diagnosis (13). Together with our results, this suggests that these clinical features already manifest in pre-diagnostic PSP.

Anticholinergic drugs may be used to treat depression, dizziness, and urinary incontinence, all of which are common symptoms observed in patients with PSP (12–14). Previous reports have also shown that constipation and urinary symptoms are commonly observed in patients with PSP and are associated with worse disease prognosis (14, 15). This frequent prescription of anticholinergic drugs and drugs for bowel disorders/constipation to patients with pre-diagnostic PSP may be a convenient approach for managing the complexity of symptoms manifested and is likely to be a proxy for the wide extent of these symptoms in PSP cases before diagnosis. While severe autonomic symptoms are usually considered as exclusion criteria for PSP (2), these can still occur in PSP but are usually less prominent as in multiple system atrophy, for example. Still, in individual patients, the presence of autonomic symptoms in the early clinical course of PSP may pose diagnostic challenges.

Outside of the multivariate model, other clinical features associated with PSP identified using the random forests approach that were known to physicians included falls/repeated falls, dizziness/giddiness, neurogenic bowel or bladder disorders, and visual disorders (2, 13, 15), while the use of antivertigo drugs and urinary incontinence products are additional proxies for these symptoms.

Overall, this suggests that clinical features associated with PSP are commonly observed in the pre-diagnostic phase and, if recognized by general practitioners, could lead to an increase in earlier referrals to neurologists to facilitate early PSP diagnoses.

### Commonly Misinterpreted Clinical Features of Pre-diagnostic PSP

Delayed referrals or diagnoses are likely to be due to the difficulty in differentiating PSP from other more common disorders. Unsurprisingly, the most common misdiagnosis was Parkinson's disease, which was observed in more than half of the patients subsequently diagnosed with PSP. When comparing our 2 multivariate models, with and without prior Parkinson's diagnosis as a variable, we found that the use of drugs for bowel disorders/constipation but not antivertigo drugs was included in the model excluding prior Parkinson's diagnosis. Moreover, there was a lower likelihood of syncope and neurogenic bowel disorder in the model including prior Parkinson's diagnosis, which may reflect successful medical management of these conditions in patients who were likely being treated for them. Further examination of a model with PD diagnosis and syncope, showed that individuals with syncope but without a PD diagnosis were more likely to have PSP, whereas individuals with syncope and a PD diagnosis were less likely to have PSP (data not shown). Although these could be chance associations, particularly given the low prevalence of syncope reported in either the cases or controls, machine learning and logistic regression identified that syncope was important enough of all the variables considered to advance for inclusion in multivariate analysis. Our results indicate that PSP cases are also commonly associated with other neurodegenerative diseases, comprised largely of Alzheimer's disease and other non-specific diagnoses such as brain atrophy. This suggests that general practitioners were aware of the possibility of neurodegenerative diseases other than Parkinson's disease or concomitant neurologic diseases in patients with pre-diagnostic PSP. From autopsy studies, the coexistence or similarity of PSP with other neurodegenerative diseases such as Alzheimer's disease, multiple system atrophy, or corticobasal degeneration have been documented (16, 17). However, this may also be a misinterpretation of the classic PSP symptoms of akinesia rigidity. Progressive supranuclear palsy also appeared to be commonly misdiagnosed as vestibular disorders, which is likely a misinterpretation of postural instability in patients with PSP. As these symptoms are clearly indicative of PSP from a neurologist's perspective, this suggests a disconnect between the terms used by general practitioners and neurologists. Therefore, our results may help to bridge the gap between referrals by helping neurologists understand how PSP symptoms are interpreted in primary care. General practitioners should be encouraged to consider overall patterns of clinical features based on our multivariate model to recognize PSP and exclude Parkinson's and other neurodegenerative diseases.

### Novel Clinical Features Associated With Pre-diagnostic PSP

The pre-existing medical conditions we identified were largely consistent with the most common diagnoses recorded by general practitioners [Parkinsonism, balance disorders (of unknown cause), stroke, depression, blackouts causing falls, and dementia] before referring a patient with PSP to a specialist in a previous study that did not include matched controls (9). Using our unbiased approach in comparing PSP cases and matched controls, we identified additional clinical conditions reported in general practice that were at least 2-fold higher in patients with pre-diagnostic PSP, including cerebrovascular disease, diabetes, and sleep disorders, which may not have been previously considered to be related to PSP by treating physicians.

Our results present the first evidence that PSP may be associated with diabetes mellitus. There is increasing evidence that diabetes mellitus plays a role in enhancing neurodegeneration by accelerating inflammatory and oxidative stress processes, particularly for Alzheimer's and Parkinson's diseases (18, 19). From experimental, clinical, and community-based epidemiologic studies, diabetes mellitus has been known to cause macro- and microstructural changes contributing to structural and functional brain pathology (20). Previous reports have suggested that diabetes mellitus may be a pre-disposing factor in tau-mediated neurodegeneration in animal models for Alzheimer's disease, in which insulin resistance leads to hyperphosphorylation of tau and thus the destabilization of microtubules contributing to synaptic and neuronal degeneration (21). Another report has shown that glucose deficiency in the brain leads to tau pathology and synaptic dysfunction (22), indirectly suggesting that lower brain glucose levels caused by lower insulin levels in diabetes mellitus may also lead to tau pathology. Conversely, tau deletion has been reported to promote brain insulin resistance (23), creating a negative feedback loop. As PSP is also mediated by tau pathology, we hypothesize that diabetes mellitus could act as a modifiable risk factor and etiologic factor for PSP: patients with diabetes mellitus have reduced brain glucose levels, which leads to less oxidative phosphorylation, increased levels of reactive oxygen species, contributing to tau pathology and a higher risk of PSP. However, this remains to be determined in future studies.

We also found a higher prevalence of cerebrovascular disease, which mainly included non-specific diagnoses relating to transient cerebral ischemic attacks and cerebral ischemia not related to hemorrhage or trauma, in PSP cases relative to controls. Vascular comorbidities are common in Alzheimer's disease and may be in part related to tau hyperphosphorylation and pathology associated with ischemia (24, 25). We speculate that cerebrovascular disease may be another possible modifiable risk factor for PSP, as such vascular factors may pose a risk to accentuating PSP 4-repeat tau damage by microvascular co-pathology (26). Therefore, increasing the compensatory cerebral reserve may help slow down the PSP neurodegeneration process.

### Limitations

The authors recognize that there are some limitations of this study. As we used a single database of EMRs from clinics in Germany with a limited number of patients with PSP, our results may lack generalizability. Indeed, the findings require future validation with other databases before they can be generalized to patients with PSP from other geographic regions. Data from EMRs offer a cost-efficient way of identifying novel prognostic and etiologic markers that can be examined in prospective epidemiology studies of rare diseases. Electronic medical records may not contain a full list of potential clinical disease manifestations, although this was unlikely in this case as the EMR we used was the basis for national medical care reimbursement. We also further adjusted for this potential bias by including medication prescriptions.

The diagnosis of PSP in this dataset was based on a clinical diagnosis by an internist or general practitioner followed by a confirmation from a neurologist. Unfortunately, the use of this retrospective EMR data did not contain pathological confirmation of PSP which can only currently be obtained post-mortem. We acknowledge this limitation of the data, and again caution that our findings should be validated in other populations and in other studies, and encourage the inclusion of pathologic information and autopsy findings from the EMR.

This study focused on identifying the pre-diagnostic differences in patients with and without PSP, but was not designed to evaluate differences between patients with PSP and Parkinson's or other neurodegenerative diseases. Given the common misdiagnoses between these disorders observed in this study, this area warrants further investigation, potentially using the methods from the present study.

While the many-to-one approach for case-matched controls may help to maximize information associated with rare diseases, it is possible that we missed some important clinical features due to the large difference in sample size (152 cases and 3,122 controls). Moreover, some of the features identified using unbiased machine learning techniques may not be clinically relevant, such as erectile dysfunction, which has a very low prevalence in both PSP cases and controls. Therefore, we used clustered logistic regression analyses adjusted for sample size to obtain the final set of features. Care to not over-fit the models could also have prevented the identification of important features. The use of random forests to assess and pre-select model variables mitigated this, allowing the model variables of potential importance to emerge consistently.

For these reasons, we understand our results do not propose a gold standard for diagnosis, but rather an approach for illustrating how selecting and incorporating features from EMRs could enhance our understanding of how pre-diagnostic PSP cases are perceived in general practice and identify novel risk factors or etiologic factors associated with early PSP.

### Conclusions and Future Perspectives

Here we present a novel approach to identify clinical features associated with pre-diagnostic PSP by applying random forests algorithms and logistic regression analyses. Based on EMRs from German clinical practices, we identified 9 clinical features that collectively indicated a high likelihood of subsequent PSP diagnosis and may aid general practitioners in recognizing early PSP. These features included 2 new possible modifiable risk factors, diabetes mellitus, and cerebrovascular disease, for further investigation to expand our understanding of the etiology of PSP.

We invite other researchers to validate our approach with other databases and observational studies. Our approach could be applied to EMRs from larger and international databases to validate our results, as well as to identify new modifiable risk factors or etiologic factors for PSP, novel pre-diagnostic features to differentiate PSP from other neurodegenerative disorders, and potential regional variations in early PSP detection in general practice. In the future, our methods could potentially serve as the basis for developing a scoring methodology for screening and diagnosis tools for PSP, as well as other rare diseases, from the perspective of general practitioners to support earlier referrals to specialists. Upon further validation, these tools may even be developed into an electronic decision-support system to alert physicians to a possible diagnosis of PSP for confirmatory referral to a neurologist.

## Data Availability Statement

The data analyzed in this study is subject to the following licenses/restrictions: The data are no longer available from the authors but can be licensed from IQVIA. Requests to access these datasets should be directed to IQVIA: https://www.iqvia.com/de-de/solutions/real-world-evidence/real-world-data-and-insights.

## Ethics Statement

Ethical review and approval was not required for the study on human participants in accordance with the local legislation and institutional requirements. Written informed consent for participation was not required for this study in accordance with the national legislation and the institutional requirements.

## Author Contributions

All authors contributed to the study concept/design, data acquisition, analysis, interpretation, as well as the writing and critical review of the manuscript, and provided final approval of the submitted version.

## Conflict of Interest

MJK has received honoraria from KAI Research in conjunction with the National Institutes of Health Award R01-AR071057, and consulting fees from AbbVie and Actualize Therapy, LLC. DMO and JZ are full-time employees of AbbVie and may hold stock and/or stock options in AbbVie. RD is a contractor with AbbVie. GUH has ongoing research collaborations with Prothena; has served as a consultant for AbbVie, Alzprotect, Asceneuron, Biogen, Biohaven, Lundbeck, Novartis, Roche, Sanofi, and UCB; has received honoraria for scientific presentations from AbbVie, Biogen, Bristol Myers Squibb, Roche, Teva, UCB, and Zambon; has received research support from CurePSP, the German Academic Exchange Service (DAAD), the German Ministry of Education and Research (BMBF), the German Parkinson's Disease Foundation (DPG), the German PSP Association (PSP Gesellschaft), the German Research Foundation (DFG), International Parkinson's Funds (IPF), VolkswagenStiftung/Lower Saxony Ministry for Science/Petermax-Müller Foundation (Etiology and Therapy of Synucleinopathies and Tauopathies); and has received institutional support from the German Center for Neurodegenerative Diseases (DZNE). The authors declare that this study received funding from AbbVie Inc. The funder contributed to the study design, research, and interpretation of the data, and to the writing, review, and approval of the publication. AbbVie also provided funding to IQVIA for licensing of the electronic medical records database sourced by the IMS® Disease Analyzer and funding for editorial support.
