# Supplementary material for: Clinical Features Observed in General Practice Associated With the Subsequent Diagnosis of Progressive Supranuclear Palsy
Source: Front Neurol. 2021 Apr 22;12:637176. doi: 10.3389/fneur.2021.637176 (PMC8100604; doi:10.3389/fneur.2021.637176)
Supplement: Supplementary file 1 [file Data_Sheet_1.docx]

**SUPPLEMENTARY MATERIAL**

| **SUPPLEMENTARY TABLE 1 │** Selection steps for PSP cases and matched controls from electronic medical records. | | |
| --- | --- | --- |
| **Selection step** | **Cases** | **Controls** |
| 1. Total available in database | 226 | 18,000 |
| 1. Age ≥40 years at PSP diagnosis | 224 | 18,000 |
| 1. Confirmed PSP diagnosis | 201 | NA |
| 1. No PSP prior to 2010 | 189 | NA |
| 1. At least 2 observations   Observation period >1 day before PSP diagnosis | 154 | 13,665 |
| 1. Analytic dataset deriving from matching on:  - Year of 1^st^ activity - Age group - Region of Germany - Duration of observation in database | 152 | 3,122 |

*NA, not applicable; PSP, progressive supranuclear palsy*

| **SUPPLEMENTARY TABLE 2 │** Characteristics of PSP cases and matched controls. | | |
| --- | --- | --- |
| **Characteristic** | **PSP cases (*n* = 152)** | **Controls (*n* = 3,122)** |
| Age, mean ± SD (min, max) | 73.9 ± 7.9 (43, 93) | 72.7 ± 9.2 (38, 97) |
| Sex, female (%) | 75 (49.3) | 1,712 (54.8) |
| Region of Germany, West (%) | 130 (85.5) | 2,865 (91.8) |
| Average time (years) in database prior to index date + SD (min, max) | 5.9 ± 6.2 (1-23) | 7.5 ± 6.1 (1-26) |
| Prior hospitalizations, *n* (%)  None  1  2  ≥ 3 | 101 (66.4)  22 (14.5)  8 (5.3)  21 (13.8) | 2,537 (81.3)  337 (10.8)  129 (4.1)  119 (3.8) |

*PSP, progressive supranuclear palsy; SD, standard deviation.*


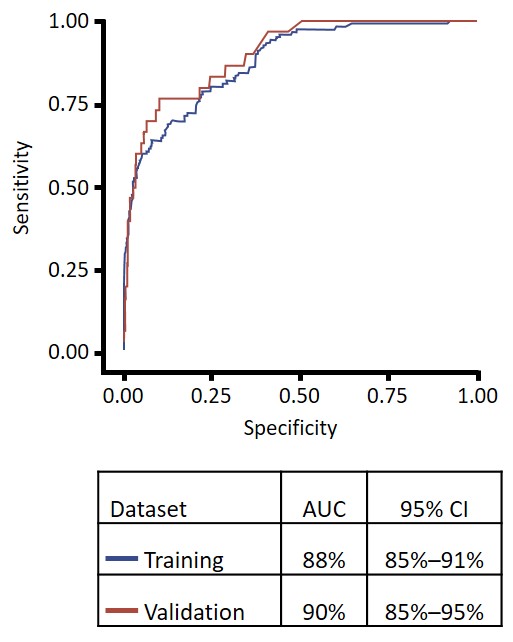
(**A**)


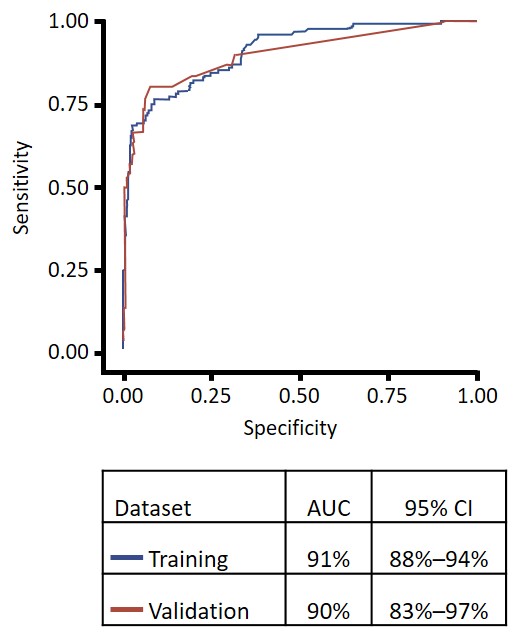
**(B)**

**SUPPLEMENTARY FIGURE 1 │** Receiver operator characteristic curves for training and validation multivariate models. **(A)** Model 1 (prior Parkinson’s diagnosis excluded as a variable; 9 variables). **(B)** Model 2 (prior Parkinson’s diagnosis included as a variable; 12 variables).
*AUC, area under the curve; CI, confidence interval.*
